# Obsessive-compulsive disorder after long-term cannabis use – case report

**DOI:** 10.1192/j.eurpsy.2023.1369

**Published:** 2023-07-19

**Authors:** D. E. Nistor, M. Corban, L. Horosan

**Affiliations:** Psychiatry, Prof. Dr. Alexandru Obregia Psychiatry Hospital, Bucharest, Romania

## Abstract

**Introduction:**

Obsessive-compulsive disorder (OCD) is characterised by intrusive thoughts and repetitive behaviours that considerably impact general functioning. Recent evidence links the endocannabinoid system to OCD neurobiology, and several case reports describe significant improvement after using dronabinol (synthetic tetrahydrocannabinol) in patients with severe OCD. Nevertheless, to what extent this new information can change our perspective on pharmacological treatment in OCD is unclear.

**Objectives:**

We present the case of a patient with obsessive-compulsive symptoms triggered after increased long-term cannabis use. Our purpose is to emphasise the necessity of continuous research and a better understanding of the correlation between OCD and cannabis derivates before formulating treatment recommendations.

**Methods:**

We used psychiatric assessments to evaluate the patient’s symptoms and evolution over time and exclude other possible causes that could have triggered the disorder.

**Results:**

Our patient is a 37-year-old man who has been frequently brought to the hospital by the police in the last 11 years for psycho-motor agitation after cannabis use. This year, he came to the hospital by himself, complaining about intrusive thoughts that required motor and mental repetitions to reduce anxiety. His obsessions were mainly about the need for symmetry and exactness and his checking compulsions about his mother’s health. The symptoms required more than half a day and caused functional impairment. A detailed history did not outline any obsessive-compulsive symptoms before the previous year. The patient denies using new drugs, and we did not identify other medical conditions that could better explain the symptoms. However, he admits to increasing the doses and frequency of cannabis use during the last year. After two weeks of cannabis abstinence and Sertraline treatment, his symptomatology improved significantly, with a reduction of more than 50% in the time spent daily on mental and motor compulsions, reduced anxiety, and a noticeable increase in overall functionality. In addition, the Yale-Brown Obsessive Compulsive Scale result decreased from 35 on the first day to 17 on discharge.

**Conclusions:**

Recent studies support the use of cannabis derivates for treating OCD symptoms. However, this case report outlines that prolonged cannabis use could also trigger OCD. Therefore, further studies are necessary to identify not only the potential benefits but also the potential risks of using cannabinoids as a pharmacological intervention.

**References:**

Nicolini H et.al.CannabisUseInPeopleWithOC.FrontPsychiatry.2021May10; 12:664228.doi:10.3389/fpsyt.2021.664228.PMID:34040556;PMCID:PMC8141625

KayserR.R. et.al.TheEndocannabinoidSystem:ANewTreatmentTargetforOCD? CannabisandCannabinoidResearch.Jun2019.7787.doi10.1089/can.2018.0049

GoodmanWK et.al.1989.YBOCS:Archives of General Psychiatry,1006–1011.doi10.1001/archpsyc.1989.01810110048007

**Disclosure of Interest:**

None Declared

